# Influencing factors of the participation of patients with severe mental disorders in community health service management

**DOI:** 10.3389/fpubh.2025.1424399

**Published:** 2025-01-17

**Authors:** Shiming Li, Yingying Ji, Queping Yang, Ying Jiang, Qitao Yin, Haohao Zhu

**Affiliations:** Affiliated Mental Health Center of Jiangnan University, Wuxi Central Rehabilitation Hospital, Wuxi, Jiangsu, China

**Keywords:** severe mental disorders, community health service management, influencing factors, Wuxi, China

## Abstract

**Objective:**

This study aimed to explore the participation of patients with severe mental disorders in community health service management and its influencing factors in Wuxi.

**Methods:**

The data of patients with severe mental disorders were extracted from the Jiangsu Provincial Management System between 2019 and 2021. The participation of patients in community health service management was analyzed. A univariate stratified analysis will be used to assess the impact of various factors on inclusion in community management. Logistic regression modeling will be employed to identify influencing factors. Ultimately, negative factors affecting participation in community health management will be identified, followed by targeted qualitative analysis through interviews.

**Results:**

A total of 27,977 individuals were included in the analysis, with 25,744 participants enrolled in community health service management, resulting in a management rate of 92.02%. Multivariate logistic regression analysis revealed the following negative factors influencing participation in community health service management: Age group 31–40 years (OR = 0.532, 95% CI: 0.434–0.653), Age group 41–50 years (OR = 0.557, 95% CI: 0.468–0.663), Age group 51–60 years (OR = 0.746, 95% CI: 0.635–0.877), Age group >60 years (OR = 0.840, 95% CI: 0.719–0.969), Duration of illness 6–10 years (OR = 0.376, 95% CI: 0.307–0.461), Duration of illness 11–15 years (OR = 0.312, 95% CI: 0.249–0.390), Duration of illness 16–20 years (OR = 0.702, 95% CI: 0.529–0.932), Duration of illness >20 years (OR = 0.701, 95% CI: 0.514–0.957), Education level of college or above (OR = 0.719, 95% CI: 0.560–0.923), Current student status (OR = 0.565, 95% CI: 0.419–0.760), Absence of social welfare benefits (OR = 0.643, 95% CI: 0.572–0.723), History of violent or harmful behavior (OR = 0.553, 95% CI: 0.442–0.692), Non-participation in community rehabilitation activities (OR = 0.170, 95% CI: 0.101–0.284). Conversely, the following factors were identified as positive influences on participation: Being a farmer (OR = 1.423, 95% CI: 1.215–1.665), Widowhood (OR = 2.355, 95% CI: 1.583–3.503), Divorce (OR = 2.992, 95% CI: 2.093–4.278). Qualitative analysis indicated that stigma associated with the illness, concerns over information disclosure, lack of social support, impacts on family members (e.g., children's education or marriage prospects), perceived ineffectiveness of community services, lack of awareness about available community health services, and the belief that services were unnecessary contributed to non-participation in community health service management.

**Conclusion:**

The participation rate of patients with severe mental disorders in health service management is relatively low in the Wuxi. Special attention should be given to patients with severe mental disorders who exhibit negative factors, such as being middle-aged or young, having a long duration of illness, a history of violent or harmful behavior, higher education levels (college and above), student status, or lack of social welfare benefits. Efforts should focus on providing social support activities, reducing stigma associated with the illness, and further improving the management rate.

## 1 Introduction

Mental health is a major public health issue in China that has been gaining increasing attention as society develops. In July 2009, the Ministry of Health, Ministry of Finance, and other departments jointly issued the “Opinions on Promoting the Gradual Equalization of Basic Public Health Services,” which included the service management of patients with severe mental disorders as one of the public health service projects in China. According to the requirements of the “Management and Treatment Guidelines for Severe Mental Disorders (2018 edition)” (1) and the “National Basic Public Health Service Specifications (Third Edition)” (2) community primary healthcare institutions are required to provide basic public health services to all severe mental disorder patients who meet the six diagnostic categories (schizophrenia, intellectual disability with comorbid mental disorders, bipolar affective disorder, schizoaffective disorder, paranoid schizophrenia, and mental disorders caused by epilepsy) and give informed consent. The specific services include patient follow-up management, risk assessment, medication guidance, rehabilitation treatment, and nursing health education.

Patients with severe mental disorders often have a high risk of relapse and disability. Therefore, effective monitoring of patients is essential to prevent disease relapse, accidents, and situations where the lack of family and community healthcare institution management can result in harm to oneself or others. Disease relapse not only affects the recovery of patients but also increases the burden on patients and society. In some cases, it can seriously disrupt public order and social stability. Therefore, it is crucial to provide effective health service management for patients with severe mental disorders in the community, including disease assessment, health education, and other basic public health services. This not only helps prevent and control disease relapse but also enables targeted interventions for specific situations (3).

The prevention and control of community mental health in China began in 1958 (4, 5). Although the development of community mental health prevention and control work was slow in the 19th century, it is widely regarded as the best approach to providing mental health treatment and care (6). Since 2004, due to the growing importance of mental health, China implemented a mental health program known as the “686 Program” (7), establishing a community mental health service system. This program promoted community mental health work in China and became a significant part of the subsequent development of community mental health services.

The community mental health service system primarily involves general practitioners and rural doctors in the early detection, treatment, and relapse prevention of patients with severe mental disorders, as well as patient information management and follow-up visits conducted at least four times a year for stabilized patients. In cases of emergencies or severe medication side effects, free crisis management services are also provided. Nurses are rarely involved in community mental health work. They assist in recording patient follow-up information and help doctors with health education for patients, such as medication guidance and management. By the end of 2020, there were 6.43 million registered patients receiving community mental health services, with a reported prevalence rate of 0.46%; 6.11 million of these patients were under management, resulting in a management rate of 95.12% (8). However, due to various reasons such as stigma, limited accessibility, and the perceived benefits of community services, the utilization rate of community mental health services in China remains low.

In terms of community mental health prevention strategies, apart from providing routine mental health promotion activities for community residents, the main prevention strategy lies in promptly identifying patients with severe mental disorders through community health services and guiding them to specialized mental health institutions for diagnosis and treatment. For registered patients with severe mental disorders in the community, community doctors are required to conduct regular follow-ups to assess their mental state and living conditions, detect signs of relapse or new mental health issues in a timely manner, and provide appropriate interventions. They also offer psychological support to patients and their families, improving their understanding of mental illness. Rehabilitation institutions or services are established in some communities to provide rehabilitation training for mental health patients, enhancing their sense of social belonging and promoting social integration. Although the quality and utilization rate of community mental health services in China remain relatively low, they still serve as the main prevention and control system for community mental health, playing a critical role.

Regarding the cost-benefit analysis of community mental health prevention and control, although certain human, material, and financial resources are required during implementation, the benefits of reducing the prevalence of mental illness, improving patients' health, reducing relapse rates, facilitating patients' reintegration into the community, and promoting social harmony outweigh the costs. These efforts not only alleviate the economic and psychological burden on patients and their families but also save substantial medical and social costs for society (9), contributing to sustainable social development. At the same time, it should be noted that mental health resources are still insufficient to meet the needs of all mental illness patients in current public health planning and resource allocation. Greater attention should be paid to and efforts made to strengthen community mental health prevention and control work, continuously optimizing prevention and control models and resource utilization efficiency to achieve greater cost-effectiveness.

## 2 Materials and methods

### 2.1 Data source

The data were obtained from the Jiangsu Provincial Management System for Severe Mental Disorders, covering the period from 2019 to 2021, and specifically focusing on registered patients with severe mental disorders in Wuxi city. Severe mental disorder patients were diagnosed according to the ICD-10 criteria and classified into six categories, including schizophrenia, intellectual disability with comorbid mental disorders, bipolar affective disorder, schizoaffective disorder, paranoid schizophrenia, and mental disorders caused by epilepsy. A total of 27,977 cases were included as study subjects, and their personal basic information and follow-up records were collected. Inclusion criteria for the study subjects were: (1) age ≥ 14 years; (2) clear diagnosis and reporting unit; (3) informed consent was obtained from guardians. Cases with a significant amount of missing basic information and those who had been transferred out of the follow-up management system were excluded. This study was approved by the ethics committee of our institution.

### 2.2 Survey content and definitions

A cross-sectional study design was used to collect and summarize the basic information and treatment details of registered patients. This included gender, age, education level, occupation, marital status, social welfare status, medical insurance, diagnostic classification, disease duration, duration of medication, and other medical information. Additional information collected included past symptoms of the patients, participation in basic public health projects for community management, community rehabilitation services, and occurrence of harmful incidents (or risk assessment level 3 or above). The determination of harmful incidents was cross-checked and confirmed with the local public security bureau. Social welfare refers to the minimum livelihood guarantee system, which provides subsidies to families with severe disabilities or loss of labor capacity due to illness. Harmful incidents are defined as violations of the Public Security Administration Punishment Law of the People's Republic of China that do not constitute a criminal offense, while harmful events are criminal behaviors that violate relevant articles of the Criminal Law of China (7). The risk assessment is divided into levels 0–5: Level 0: No behavior matching any of the criteria for levels 1–5. Level 1: Verbal threats or shouting, but no destructive behavior. Level 2: Destructive behavior confined to the home, targeting property, and can be stopped through persuasion. Level 3: Significant destructive behavior, indiscriminate of location, targeting property, and cannot be stopped through persuasion. Level 4: Persistent destructive behavior, indiscriminate of location, targeting property or people, and cannot be stopped through persuasion. Level 5: Violent behavior targeting individuals with controlled dangerous weapons, or actions such as arson or explosions. We reviewed the risk assessment levels (0–5) of patients in the archival system and defined levels 3 and above as “violent or harmful behavior.”

### 2.3 Statistical analysis

Data analysis was conducted using SPSS 19.0 statistical software. Continuous data were presented as X ± s, while categorical data were tested using the chi-square test. Group comparisons were performed using the chi-square test. Variables with statistically significant differences were included in the multivariable logistic regression analysis. The factors influencing the participation of patients in community health service management were analyzed through regression analysis, with the assignment of independent variables as shown in Table 1. A significance level of *P* < 0.05 was considered statistically significant. In the qualitative study, we used a convenience sampling method to randomly select some patients who declined community management for interviews. The interviews were conducted with open-ended questions, such as “Why are you unwilling to participate in community management?” to understand the true thoughts and reasons behind patients' refusal to participate in community management.

**Table 1:** Assignment of variables for logistic regression.

**Variable**	**Assignment**
Participation in community health service management	0 = No, 1 = Yes
Age (years)	0 = ≤ 18, 1 = 19–30, 2 = 31–40, 3 = 41–50, 4 = 51–60, 5 = >60
Education level	0 = Primary school and below, 1 = Junior high school, 2 = High school/vocational school, 3 = College and above
Occupation	0 = Unemployed or jobless, 1 = Employed, 2 = Farmer, 3 = Student, 4 = Retired, 5 = Professional/technical personnel
Registered residence	0 = Local, 1 = Non-local
Marital status	0 = Unmarried, 1 = Married, 2 = Widowed, 3 = Divorced
Violent or harmful behavior	0 = No, 1 = Yes
Social welfare status	0 = No, 1 = Yes
Disease duration (years)	0 = 0–5, 1 = 6–10, 2 = 11–15, 3 = 16–20, 4 = >20
Duration of medication (years)	0 = 0–5, 1 = 6–10, 2 = 11–15, 3 = 16–20, 4 = >20
Disability certificate status	0 = None, 1 = Mental disability certificate, 2 = Physical disability certificate
Participation in community rehabilitation activities	0 = Yes, 1 = No

## 3 Results

### 3.1 Demographic characteristics

A total of 27,977 patients were included in the study, among which 25,744 patients agreed to participate in community management. Of the patients who agreed to participate, 12,159 were male and 13,585 were female, with an average age of 48.57 ± 12.35 years. The majority had an education level of elementary school or below (11,543 patients). The most common occupation was unemployed or laid-off workers (8,308 patients). Regarding marital status, the highest proportion were married (14,200 patients), and the majority were economically non-poor (19,463 patients). The average duration of illness was 15.02 ± 5.24 years, and the average duration of medication use was 16.01 ± 3.99 years.

A total of 2,233 patients declined to participate in community management. Among them, 1,043 were male and 1,190 were female, with an average age of 44.77 ± 13.64 years. The majority had an education level of elementary school or below (789 patients). The most common occupation was unemployed or laid-off workers (712 patients). Regarding marital status, the highest proportion were married (1,223 patients), and the majority were economically non-poor (1,726 patients). The average duration of illness was 14.23 ± 3.81 years, and the average duration of medication use was 15.10 ± 4.20 years. As shown in Table 2.

**Table 2:** General information of included patients.

**Factors**	**Total number**	**Agreed to participate (*n* = 25,744)**	**Declined to participate (*n* = 2,233)**
**Gender**			
Male	13,202	12,159 (92.10%)	1,043 (7.90%)
Female	14,775	13,585 (91.95%)	1,190 (8.05%)
Age	27,969	48.57 ± 12.35	49.77 ± 13.64
**Education level**			
Primary school and below	12,332	11,543 (93.60%)	789 (6.40%)
Junior school	9,597	8,894 (92.67%)	703 (7.33%)
High/vocational school	3,825	3,437 (89.86%)	388 (10.14%)
College and above	2,016	1,682 (83.43%)	334 (16.57%)
**Occupation**			
Unemployed or jobless	9,020	8,308 (92.11%)	712 (7.89%)
Employed	3,560	3,194 (89.72)	366 (10.28%)
Farmer	7,319	6,887 (94.10)	432 (5.90%)
Student	459	366 (79.74%)	93 (20.26%)
Retired	3,153	2,965 (94.04%)	188 (5.96%)
Professional/technical personnel	328	271 (82.62%)	57 (17.38%)
**Registered residence**			
Local	27,808	25,606 (92.08%)	2,202 (7.92%)
Non-local	169	138 (81.66%)	31 (18.34%)
**Marital status**			
Unmarried	9,234	8,485 (91.89%)	749 (8.11%)
Married	15,423	14,200 (92.07%)	1,223 (7.93%)
Widowed	1,055	984 (93.27%)	71 (6.73%)
Divorced	1,919	1,796 (93.59%)	123 (6.41%)
**Violent or harmful behavior**			
Yes	2,008	1,916 (95.42%)	92 (4.58%)
No	25,969	23,828 (91.76%)	2,141 (8.24%)
**Economic status**			
Impoverished	6,788	6,281 (92.53%)	507 (7.47%)
Non-impoverished	21,189	19,463 (91.85%)	1,726 (8.15%)
**Social welfare status**			
Yes	4,858	4,556 (93.78%)	302 (6.22%)
No	23,119	21,188 (91.65%)	1,931 (8.35%)
**Disability certificate status**			
Yes	15,368	13,631 (88.70%)	1,737 (11.30)
Mental disability certificate	8,266	7,881 (95.34%)	385 (4.66%)
Physical disability certificate	4,251	4,141 (97.41%)	110 (2.59%)
**Participation in community rehabilitation activities**			
No	1,240	1,225 (98.79%)	15 (1.21%)
Yes	26,737	24,519 (91.70%)	2,218 (8.30%)
Disease duration (years)	27,977	15.02 ± 5.24	16.01 ± 3.99
Medication duration (years)	27,973	14.23 ± 3.81	15.10 ± 4.20

### 3.2 Community health service management participation among patients with severe mental disorders of different characteristics

The participation rate of severe mental disorder patients in community health service management was 92.02%. There were significant differences in the participation rate among different age groups, education levels, occupations, marital statuses, disease durations, and durations of medication (*P* < 0.05). Patients with local registered residence had a higher participation rate compared to non-local patients. Patients with violent or harmful behavior had a higher participation rate compared to those without a history. Patients receiving social welfare benefits had a higher participation rate compared to those without benefits. Patients with a disability certificate had a higher participation rate compared to those without a certificate. Patients who did not participate in community rehabilitation activities had a higher participation rate compared to non-participants. These differences were statistically significant (*P* < 0.05). Refer to Table 3 for details.

**Table 3:** Stratified analysis of factors affecting community management participation.

**Factors**	**Total number**	**Participants**	**Rate (%)**	***χ^2^***	***P***
**Gender**					
Male	13,202	12,159	92.10	0.23	0.64
Female	14,775	13,585	91.95		
**Age**					
≤ 18	368	344	93.48	260.74	< 0.05
19 30	2,780	2,392	86.04		
31 40	4,352	3,873	88.99		
41 50	5,820	5,386	92.54		
51 60	6,494	6,068	93.44		
>60	8,155	7,677	94.14		
**Education level**					
Primary school and below	12,332	11,543	93.60	287.67	< 0.05
Junior school	9,597	8,894	92.67		
High/vocational school	3,825	3,437	89.86		
College and above	2,016	1,682	83.43		
**Occupation**					
Unemployed or jobless	9,020	8,308	92.11	229.82	< 0.05
Employed	3,560	3,194	89.72		
Farmer	7,319	6,887	94.10		
Student	459	366	79.74		
Retired	3,153	2,965	94.04		
Professional/ technical personnel	328	271	82.62		
**Registered residence**					
Local	27,808	25,606	92.08	24.86	< 0.05
Non-local	169	138	81.66		
**Marital status**					
Unmarried	9,234	8,485	91.89	70.01	< 0.05
Married	15,423	14,200	92.07		
Widowed	1,055	984	93.27		
Divorced	1,919	1,796	93.59		
**Violent or harmful behavior**					
Yes	2,008	1,916	95.42	34.05	< 0.05
No	25,969	23,828	91.76		
**Economic status**					
Impoverished	6,788	6,281	92.53	3.21	0.07
Non-impoverished	21,189	19,463	91.85		
**Social welfare status**					
Yes	4,858	4,556	93.78	24.94	< 0.05
No	23,119	21,188	91.65		
**Disease duration (years)**					
0 5	2,730	2,322	85.05	435.88	< 0.05
6 10	4,184	3,666	87.62		
11 15	3,922	3,574	91.13		
16 20	3,570	3,329	93.25		
>20	13,571	12,853	94.71		
**Medication duration (years)**					
0 5	9,136	8,230	90.08	249.09	< 0.05
6 10	4,373	3,875	88.61		
11 15	3,449	3,177	92.11		
16 20	2,976	2,797	93.99		
>20	8,039	7,661	95.30		
**Disability certificate status**					
Yes	15,368	13,631	88.70	529.50	< 0.05
Mental disability certificate	8,266	7,881	95.34		
Physical disability certificate	4,251	4,141	97.41		
**Participation in community rehabilitation activities**					
No	1,240	1,225	98.79	81.02	< 0.05
Yes	26,737	24,519	91.70		

### 3.3 Influencing factors of the participation of patients with severe mental disorders in community health service management

The variables with statistical significance in the stratified analysis were included in a multivariate logistic regression model. The results indicated that the main factors influencing participation in community health service management among patients with severe mental disorders include age, duration of illness, marital status, education level, presence of social welfare benefits, occupation, history of violent or harmful behavior, and participation in community rehabilitation activities. Compared with the < 18-year-old group, patients in the 31–40, 41–50, 51–60, and >60 age groups were more likely to refuse participation in community health service management. Compared with the group with a duration of illness of 0–5 years, patients with a duration of 6–10 years, 11–15 years, 16–20 years, and >20 years were more likely to refuse participation. Patients with a college education or above, students, those without social welfare benefits, those with a history of violent or harmful behavior, and those who do not participate in community rehabilitation activities were negative factors for participation in community health service management. On the other hand, being a farmer, widowed, or divorced were positive factors for participation (see Table 4).

**Table 4:** Multivariate logistic regression analysis of factors influencing the participation of patients with severe mental disorders in community health service management.

**Factors**	**β**	**Wald χ^2^**	***P***	**OR**	**95% CI**	
					**Lower**	**Upper**
**Age**						
≤ 18				1.000		
19 30	0.364	2.119	0.145	1.439	0.881	2.350
31 40	−0.631	36.687	< 0.05	0.532	0.434	0.653
41 50	−0.585	43.011	< 0.05	0.557	0.468	0.663
51 60	−0.293	12.547	< 0.05	0.746	0.635	0.877
>60	−0.180	5.640	0.018	0.835	0.719	0.969
**Disease duration (years)**						
0 5				1.000		
6 10	−0.978	88.491	< 0.05	0.376	0.307	0.461
11 15	−1.166	102.53	< 0.05	0.312	0.249	0.390
16 20	−0.354	6.000	0.014	0.702	0.529	0.932
>20	−0.355	4.994	0.025	0.701	0.514	0.957
**Education level**						
Primary school and below				1.000		
Junior school	0.088	1.854	0.173	1.092	0.962	1.240
High/vocational school	0.020	0.051	0.821	1.020	0.860	1.209
College and above	−0.330	6.691	0.010	0.719	0.560	0.923
**Occupation**						
Unemployed or jobless				1.000		
Employed	0.048	0.335	0.563	1.049	0.892	1.233
Farmer	0.352	19.260	< 0.05	1.423	1.215	1.665
Student	−0.572	14.187	< 0.05	0.565	0.419	0.760
Retired	0.415	1.506	0.264	1.515	1.232	1.862
Professional/technical personnel	−0.069	0.167	0.683	0.933	0.671	1.299
**Marital status**						
Unmarried				1.000		
Married	0.884	2.537	0.846	2.420	1.778	3.294
Widowed	0.857	17.888	< 0.05	2.355	1.583	3.503
Divorced	1.096	36.098	< 0.05	2.992	2.093	4.278
**Social welfare status**						
Yes				1.000		
No	−0.441	54.660	< 0.05	0.643	0.572	0.723
**Violent or harmful behavior**						
No				1.000		
Yes	−0.592	27.022	< 0.05	0.553	0.442	0.692
**Participation in community rehabilitation activities**						
Yes				1.000		
No	−1.775	45.645	< 0.05	0.170	0.101	0.284

### 3.4 Qualitative study on patients who declined community management

Based on preliminary telephone and in-home interviews conducted for this study, we investigated 286 patients who declined community management. Among them, 102 patients were interviewed in person during home visits, and 184 patients were surveyed via telephone. The primary characteristics of these patients included high educational attainment and being part of a mobile population. The reasons for refusing community management were mainly composed of stigma associated with the illness, concerns about information disclosure, lack of social support, impacts on family harmony, hindrances to young people's education and marriage, dissatisfaction with the perceived outcomes of community services, lack of understanding about community health services, and the belief that services or medication were unnecessary. See Table 5 for details.

**Table 5:** Analysis of reasons for not participating in community management.

**Factors**	**Number of patients**	**Proportion (%)**
Stigma associated with the illness	215	75.26%
Concerns about information disclosure, impact on family harmony, and lack of social support	203	71.15%
Dissatisfaction with expected outcomes	178	62.11%
Hindrances to young people's education and marriage	165	57.85%
Guardians' lack of understanding about community service management	158	55.24%
Belief that medication or visits are unnecessary	147	51.48%

## 4 Discussion

Patients with severe mental disorders often experience relapses and have long-term illness durations. The majority of their recovery time is spent in the community, making community health service management particularly important for these patients. It plays a crucial role in promoting the long-term stability of patients with severe mental disorders. In China, the government has included the management and treatment of severe mental disorders in the national basic public health service program (1).

This study found that the rate of community health service management among patients with severe mental disorders in Wuxi was 92.02%, which is lower than the national rate of 95.12% (8). The level of economic development, cultural background, and assistance policies in different regions influence the community health service management of patients with severe mental disorders. There are often regional disparities in development and significant differences in the utilization of public health services (10, 11). This study provides scientific evidence to improve the management rate in the local area by analyzing the participation of patients with severe mental disorders in community health service management and its influencing factors.

Effective management of patients with severe mental disorders not only helps alleviate the economic and family burden for patients and their families but also reduces the social burden through increased social support and basic public health services (12). This study found that older patients are less willing to participate in community health service management compared to younger patients. This may be due to older patients being less aware of community health service management policies and having difficulty understanding the meaning of basic public health services, making it more challenging for them to accept such services. We also found that longer duration of illness is a negative factor for participating in community health service management. This may be because patients with longer illness durations experience repeated episodes, long-term medication, and lose hope in the treatment of their illness, leading to a negative attitude toward the disease and a lack of confidence in primary community health services (13). Patients with higher education levels, particularly those with college and above education, are also less likely to accept community health service management. This could be attributed to increased awareness and understanding of mental illnesses with higher levels of education, as well as concerns about privacy, making it more challenging for them to participate in community health service management (14). Compared to unemployed individuals, students show a negative association with participating in community health service management. This could be because students may feel protected in their school environment and may be reluctant to disclose their mental illnesses, experiencing stigma and avoiding community health service management (15). Patients who do not receive social welfare support also show a negative association with participating in community health service management. Patients who do not qualify for social welfare support often have better financial conditions and more ability and experience in taking care of themselves, making them less inclined to participate in free community health service management programs. Patients who do not participate in community rehabilitation activities also show a negative association with participating in community health service management. Compared to patients who participate in community rehabilitation activities, those who do not receive the same level of social support, including care from the neighborhood or community, and planned rehabilitation activities from mental health professionals. These factors can promote patient recovery, improve their community functioning, reduce self-stigma, alleviate the burden on families, and facilitate the acceptance of basic public health services (16, 17).

Furthermore, this study found that patients with occupations as farmers are more likely to participate in community-based health service management. This could be attributed to their limited knowledge of mental health and reliance on primary community health services, leading to better compliance with community health service management. In contrast, compared to unmarried patients, those who are widowed or divorced are more likely to participate in community health service management. This may be because patients rely more on social support after marital failure and tend to have greater trust in community health workers (18). This can help them effectively manage their mental illness, prevent relapses, and reduce the occurrence of harmful behaviors. Several studies have shown that comprehensive community management interventions for patients with severe mental disorders have positive effects, reducing relapses and harmful behaviors, and alleviating the burden on both society and patients (19, 20). Therefore, it is essential to encourage the participation of patients with severe mental disorders in basic public health service programs, promote their social functioning, and integrate them into society.

In the qualitative interviews targeting patients less likely to participate in community management, we found that 50% of individuals cited stigma, concerns about information disclosure, worries about the impact of their illness on family finances, education, and social interactions, as well as insufficient awareness of community health services, as the main reasons for not participating. It is not difficult to imagine that discrimination against mental illness is still prevalent in society. Many parents teach their children to avoid mental illness patients, highlighting the challenges of integrating patients with mental disorders into society. Furthermore, the confidentiality concerns raised by some patients and families stem from community staff collecting patient information and conducting home visits or telephone follow-ups according to guidelines, which can cause distress. Some patients also reported that receiving community health services did not improve their condition, leading to a gradual loss of interest in these services and their refusal to participate in follow-up management. Additionally, there are patients who believe their condition is stable and that reducing or discontinuing medication has no impact on their health, making them less reliant on community management.

The strengths of this study lie in its large sample size, which investigated whether patients with severe mental disorders participate in community health service management and explored the influencing factors. Additionally, qualitative interviews with patients who declined community management were conducted to understand their subjective perceptions, providing valuable insights for future mental health work. However, this study has certain limitations. First, the data were sourced from Wuxi City, making the data source singular. The exclusion of patients with incomplete information and those without a confirmed diagnosis also impacts the results, limiting the generalizability of the findings across other regions in China. Furthermore, the lack of information about patient caregivers, as well as details regarding patient satisfaction, relapse rates, and the efficiency of basic public health services after participating in community health service management, requires further exploration. Future research will adopt longitudinal designs, more detailed variable definitions, advanced statistical analyses, and additional data sources to further investigate related topics.

In conclusion, patients with severe mental disorders should be encouraged to participate in basic public health service programs to promote their recovery of social functioning and reintegration into society, while providing adequate social support. Policies supporting free medication for impoverished patients, increasing the frequency and amount of medical assistance (21), and reducing societal discrimination against patients and their families should be implemented. For patients who decline participation in community health service management, targeted health education and policy promotion should be conducted to improve the management rate of community health services for patients with severe mental disorders.

## Data Availability

The original contributions presented in the study are included in the article/supplementary material, further inquiries can be directed to the corresponding authors.
